# Supplementary material for: Risk Factors and Prognosis of Stroke in Gynecologic Cancer Patients
Source: Cancers (Basel). 2023 Oct 9;15(19):4895. doi: 10.3390/cancers15194895 (PMC10572068; doi:10.3390/cancers15194895)
Supplement: Supplementary file 1 [file cancers-15-04895-s001.zip › Table S1.pdf]

Table S1. Prognostic factors associated with progression-free survival

Variables	Univariate analysis		Multivariate analysis	
	HR (95% CI)	*P*-value	HR (95% CI)	*P*-value
Age (yr)	1.019 (1.011–1.028)	< 0.001	1.010 (1.002–1.019)	0.017
BMI (Kg/m^2)	0.948 (0.921–0.976)	< 0.001	0.965 (0.938–0.994)	0.016
DM				
No	1.00 (Reference)			
Yes	1.058 (0.771–1.452)	0.727		0.077
HTN				
No	1.00 (Reference)			
Yes	1.052 (0.825–1.342)	0.683		0.733
PTE/DVT				
No	1.00 (Reference)		1.00 (Reference)	
Yes	2.060 (1.335–3.181)	0.001	1.592 (1.016–2.494)	0.042
Heart disease[1]				
No	1.00 (Reference)			
Yes	0.969 (0.712–1.319)	0.842		
Fractures[2]				
No	1.00 (Reference)			
Yes	0.949 (0.520–1.733)	0.865		
Stroke onset				
No stroke	1.00 (Reference)			
Stroke before / after tx	1.289 (0.809–2.051)	0.285		
Stroke during tx	1.714 (0.961–3.056)	0.068		
Cancer type				
Cervix / Endometrial	1.00 (Reference)		1.00 (Reference)	
Ovary	1.946 (1.551–2.441)	< 0.001	1.080 (0.834–1.399)	0.560
Histological subtype				
Usual[3]	1.00 (Reference)		1.00 (Reference)	
Unusual	1.626 (1.264–2.091)	< 0.001	1.581 (1.215–2.058)	0.001
Histological grade				
Well/Moderate	1.00 (Reference)		1.00 (Reference)	
Poor	2.662 (1.999–3.544)	< 0.001	1.941(1.258–2.995)	0.003
Stage				
I/II	1.00 (Reference)		1.00 (Reference)	
III/IV	3.773 (3.007–4.735)	< 0.001	3.412 (2.628–4.430)	< 0.001

[1]Heart disease includes atrial fibrillation, coronary artery disease, dyslipidemia or heart failure. [2]Fractures include fractures located in the femur, fibular, patella, rib, or spine and total hip or knee replacement. [3]Usual type indicates squamous cell or adenocarcinoma in cervix, endometrioid carcinoma in endometrium, and serous or endometrioid carcinoma in ovary. Abbreviations: PFS, progression free survival; HR, hazard ratio; CI, confidence interval; BMI, body mass index; DM, diabetes mellitus; HTN, hypertension; PTE, pulmonary thromboembolism; DVT, deep vein thrombosis; Tx, treatment.
