# Supplementary figures and images for: Risk Factors and Prognosis of Stroke in Gynecologic Cancer Patients
Source: Cancers (Basel). 2023 Oct 9;15(19):4895. doi: 10.3390/cancers15194895 (PMC10572068; doi:10.3390/cancers15194895)

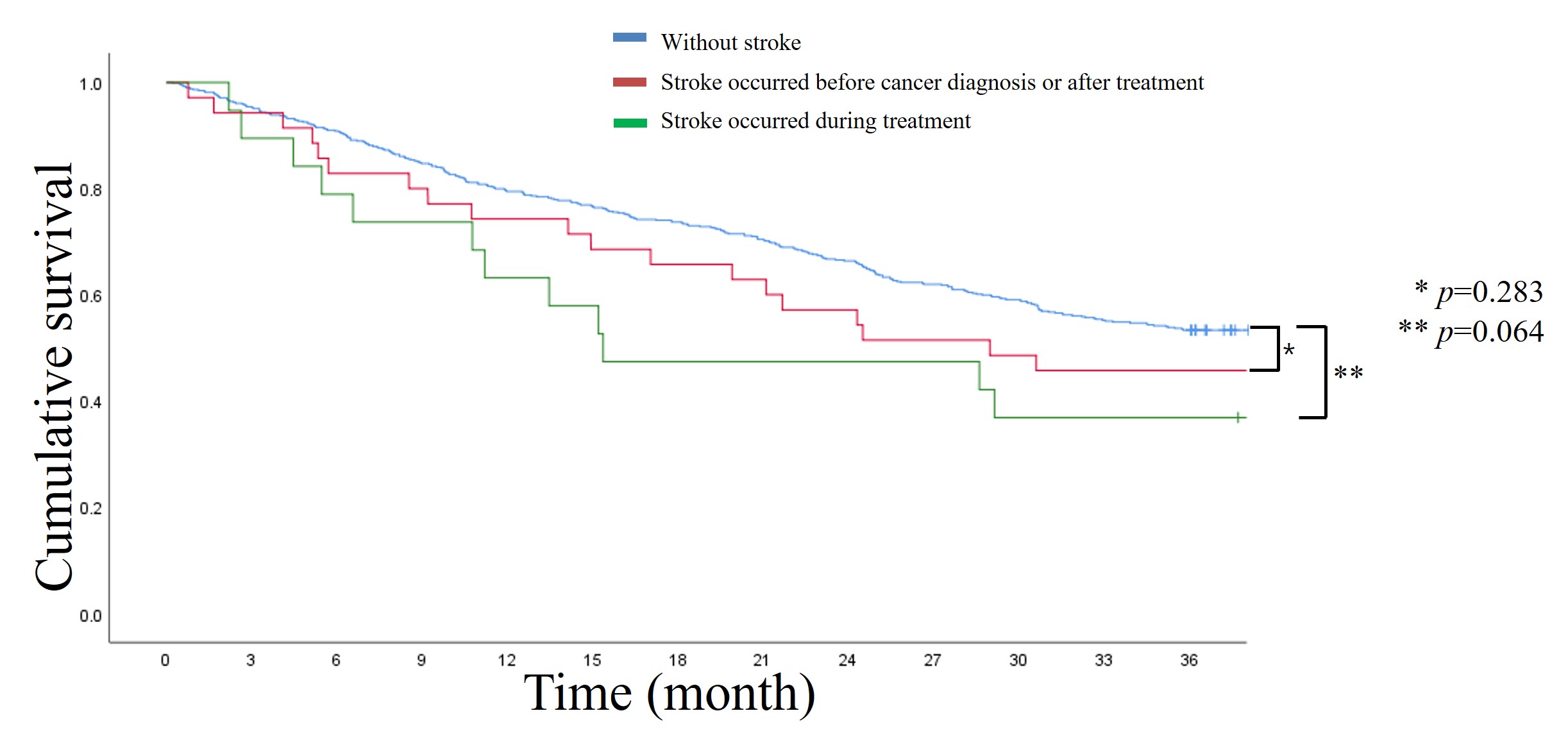

Supplement: Supplementary file 1 [file cancers-15-04895-s001.zip › Figure S1.jpg]
